# Influenza pandemic and professional duty: family or patients first? A survey of hospital employees

**DOI:** 10.1186/1471-2458-6-311

**Published:** 2006-12-28

**Authors:** Boris P Ehrenstein, Frank Hanses, Bernd Salzberger

**Affiliations:** 1 Dept. of Internal Medicine I, University Medical Center Regensburg, Germany

## Abstract

**Background:**

Conflicts between professional duties and fear of influenza transmission to family members may arise among health care professionals (HCP).

**Methods:**

We surveyed employees at our university hospital regarding ethical issues arising during the management of an influenza pandemic.

**Results:**

Of 644 respondents, 182 (28%) agreed that it would be professionally acceptable for HCP to abandon their workplace during a pandemic in order to protect themselves and their families, 337 (52%) disagreed with this statement and 125 (19%) had no opinion, with a higher rate of disagreement among physicians (65%) and nurses (54%) compared with administrators (32%). Of all respondents, 375 (58%) did not believe that the decision to report to work during a pandemic should be left to the individual HCP and 496 (77%) disagreed with the statement that HCP should be permanently dismissed for not reporting to work during a pandemic. Only 136 (21%) respondents agreed that HCW without children should primarily care for the influenza patients.

**Conclusion:**

Our results suggest that a modest majority of HCP, but only a minority of hospital administrators, recognises the obligation to treat patients despite the potential risks. Professional ethical guidelines allowing for balancing the needs of society with personal risks are needed to help HCP fulfil their duties in the case of a pandemic influenza.

## Background

Medical providers worldwide are gearing up for a likely pandemic of human influenza. Professional duty of healthcare professionals (HCP) may clash with fear of contracting influenza or its transmission to family members. Triggered by the experience responding to the SARS epidemic in 2003, the lack of official ethical guidelines on balancing public needs and personal risk has been pointed out [1]. Prompted by discussions at our institution on proper response to a pandemic influenza outbreak, we examined employees' knowledge on H5N1 (avian) influenza and solicited their opinions on professional ethics.

## Methods

In February 2006, we distributed anonymous self-administered, multiple-choice paper questionnaires to the 637 physicians and final-year medical students (FYMS), 994 nurses, and 267 hospital administrators at the university hospital in Regensburg, Germany. All questions were multiple-choice. Items (4) on knowledge about H5N1 (avian) influenza, in the 'True/False/Don't know' format, assessed awareness about general human-to-human transmissibility; the availability of a commercial vaccine; the projected efficacy of neuraminidase inhibitors; and the resistance against neuraminidase inhibitors. Further four items (Table 1), pertaining to ethical issues of influenza pandemic management, offered statements to be endorsed using a five-point scale: 'strongly disagree', 'disagree', 'no opinion', 'agree', 'strongly agree'. We also collected data on gender, age (above or below 35 years), having minor children, and professional group (as defined above). We used the χ^2^-test to assess differences between groups, using SPSS v12.0G software (SPSS Inc.). Since the study was anonymous, formal institutional review board approval was not required, but approval by the main board of the medical centre representing all professional groups was obtained before conducting the survey.

## Results

We received 644 (34%) completed surveys: 233 of 637 (37%) from physicians and FYMS, 264 of 994 (27%) from nurses, and 147 of 267 (55%) from administrators. There were no statistically significant differences among respondents and non-respondents regarding age (51% vs. 49% younger than 35 years) or gender (39% vs. 37% males). Absence of general human-to-human transmissibility of avian influenza was correctly indicated by 566 (88%) respondents, absence of commercially available anti-H5N1 influenza vaccine was known to 543 (84%) respondents, while 171 (27%) participants knew about descriptions of resistance to neuraminidase inhibitors of H5N1 strains (44% of physicians and FYMS, 17% of nurses and 16% of administrators). Surprisingly, only 65 (10%) respondents believed that prophylactic use of neuraminidase inhibitors could protect them from H5N1 influenza in the event of human-to-human transmission. Overall, 182 (28%) respondents strongly agreed or agreed that it is professionally acceptable for HCP to abandon their workplace during a pandemic in order to protect themselves and their families, 337 (52%) respondents disagreed or strongly disagreed with this, while 125 (19%) selected the 'no opinion' option (Table 1). The proportion of disagreeing respondents was 65% among physicians and FYMS, 54% among nurses, and 30% among administrators (p < .001). A majority of respondents did not believe that the decision to report to work during a pandemic should be left to the individual HCP; these proportions differed significantly among the three professional groups (Table 1, p < .001). Of the 644 respondents, 79 (12%) agreed and 496 (77%) disagreed with the statement that HCP should be permanently dismissed for not reporting to work during a pandemic. More than one-fifth (n = 136, 21%) of the respondents agreed with the statement that during a pandemic HCP without small children should primarily care for the influenza patients. This proportion was higher among female respondents with (37%) than without (16%) minor children, but was similar for male respondents regardless wether they did (22%) or did not (23%) have minor children.

## Discussion

Although issues of medical professionalism have been discussed in the aftermath of the SARS epidemic [1-8], to our knowledge, only few studies have addressed this topic in relation to the anticipated influenza pandemic [9]. Our results suggest that most HCP at our institution recognise their professional obligation to treat patients despite the potential risks. A majority of respondents disagreed that reporting to work during pandemic should be an individual decision of HCP; at the same time, most respondents would not like to see non-reporting HCP harshly punished. The latter finding may reflect the recognition of the difficulty of decisions that HCP have to face.

Although HCP generally accept their obligation to the public, personal risks involved, coupled with lack of clear ethical standards confronting an influenza pandemic, place ethical burden of decision on individual HCP. The existing general ethical guidelines of professional societies, e.g. the Amercian Medical Association [10], establish the duty to treat despite possible risks. Our findings underscore the importance of incorporating these guidelines in the pandemic preparedness plans and to foster a broader discussion among HCP about these guidelines and their implications [1,6,7,9]. For example, while acknowledging the difficulty of recruiting enough medical personnel to care for patients during a potential pandemic, German federal health authorities (Robert Koch Institute) ignore ethical issues of deciding which HCP should be assigned to highly infectious patients; similarly, the effect of fear of contracting a potential lethal influenza infection among HCP is not discussed [11]. A recently published draft of a WHO working group gives a good overview of ethical controversies in regard to the role and obligations of HCP during an outbreak of pandemic influenza and provides preliminary recommendations for pandemic planning [12].

A majority of HCP participating in this survey, including those with small children, asserted their readiness to care for patients during a pandemic. In the era of vaccines and antibiotics the actual and perceived professional risks for HCP have declined [3]. Faced with the possibility of contracting a potentially lethal disease during the SARS epidemic, some of the involved HCP questioned their choice of career and indeed some left their profession [3]. To get a better appraisal of the HCP willingness to accept professional risks, we included hospital administrators as a surrogate group for the general population in our survey. The rate of administrators not willing to accept personal risk was approximately twice as high as the rate of HCP.

Only about one third of the distributed questionnaires were returned, with nurses having the lowest response rate. If persons with perceived undesirable answers were less likely to respond, we could have over-estimated the willingness of HCP to forgo personal safety in order to care for highly infectious patients. Of course, the actual HCP behaviour during a pandemic should not solely be predicted by their answers to the hypothetical questions of a survey. Nevertheless, our study illuminates aspects of HCP perceptions of risk and duty while facing a possible influenza pandemic.

Our survey should be replicated in different healthcare settings or other countries to learn more about the generisability of the results. Regardless, we believe that the surveyed HCP at our tertiary-care, 1000-bed medical centre represent well clinicians confronting a potential influenza pandemic, because – at least in the initial phase of a pandemic – patients will be referred to tertiary care facilities for treatment.

## Conclusion

Despite potential risks, most participating HCP recognized their professional obligation to treat patients during an influenza pandemic. Morale of HCP may be bolstered by better education about projected efficacy and availability of neuraminidase inhibitors during a pandemic. Professional ethical guidelines allowing for balancing the needs of society with personal risks are needed to help HCP fulfil their duties in the case of a pandemic influenza.

## Competing interests

The author(s) declare that they have no competing interests.

## Authors' contributions

BPE designed the study, conducted the paper-based survey, analysed the data, and drafted the manuscript. FH participated in the design of the study and helped to draft the manuscript. BS participated in the design of the study, in the statistical analysis and helped to draft the manuscript. All authors read and approved the final version of the manuscript.

## Figures and Tables

**Table 1:** Opinions of health care professionals (HCP) on professional ethical topics regarding the management of an influenza pandemic stratified by profession (in percent of each stratum).

	**Physicians/FYMS**^**1 **^n = 233			**Nurses **n = 264			**Administrators **n = 147		
	**Agree**	**No opinion**	**Disagree**	**Agree**	**No opinion**	**Disagree**	**Agree**	**No opinion**	**Disagree**

It would be ethical for HCP to abandon their workplace during a pandemic in order to protect themselves and their families.	24%	11%	64%	26%	20%	54%	37%	33%	30%
HCP should be allowed to decide whether they report to work during a pandemic.	25%	8%	67%	29%	16%	55%	29%	21%	50%
HCP not reporting to work during a pandemic should be permanently dismissed.	13%	9%	79%	12%	10%	78%	12%	15%	74%
HCP without children should primarily care for influenza patients during a pandemic.	16%	12%	72%	22%	13%	65%	27%	18%	54%

Note: ^1^final-year medical students
